# Podocyte RNA sequencing reveals Wnt- and ECM-associated genes as central in FSGS

**DOI:** 10.1371/journal.pone.0231898

**Published:** 2020-04-17

**Authors:** Eva Nora Bukosza, Klaus Kratochwill, Christoph Kornauth, Helga Schachner, Christoph Aufricht, Christoph A. Gebeshuber

**Affiliations:** 1 Translational Medicine Institute, Semmelweis University Budapest, Budapest, Hungary; 2 Division of Pediatric Nephrology and Gastroenterology, Department of Pediatrics and Adolescent Medicine, Medical University of Vienna, Vienna, Austria; 3 Christian Doppler Laboratory for Molecular Stress Research in Peritoneal Dialysis, Department of Pediatrics and Adolescent Medicine, Medical University of Vienna, Vienna, Austria; 4 Clinical Division of Hematology and Hemostaseology, Department of Internal Medicine I, Medical University of Vienna, Vienna, Austria; 5 Clinical Institute of Pathology, Medical University of Vienna, Vienna, Austria; University of Crete, GREECE

## Abstract

Loss of podocyte differentiation can cause nephrotic-range proteinuria and Focal and Segmental Glomerulosclerosis (FSGS). As specific therapy is still lacking, FSGS frequently progresses to end-stage renal disease. The exact molecular mechanisms of FSGS and gene expression changes in podocytes are complex and widely unknown as marker changes have mostly been assessed on the glomerular level. To gain a better insight, we isolated podocytes of *miR-193a* overexpressing mice, which suffer from FSGS due to suppression of the podocyte master regulator *Wt1*. We characterised the podocytic gene expression changes by RNAseq and identified many novel candidate genes not linked to FSGS so far. This included strong upregulation of the receptor tyrosine kinase *EphA6* and a massive dysregulation of circadian genes including the loss of the transcriptional activator *Arntl*. By comparison with podocyte-specific changes in other FSGS models we found a shared dysregulation of genes associated with the Wnt signaling cascade, while classical podocyte-specific genes appeared widely unaltered. An overlap with gene expression screens from human FSGS patients revealed a strong enrichment in genes associated with extra-cellular matrix (ECM) and metabolism. Our data suggest that FSGS progression might frequently depend on pathways that are often overlooked when considering podocyte homeostasis.

## Introduction

Focal and Segmental Glomerulosclerosis (FSGS) is a clinico-pathological syndrome referring to sclerotic lesions in some areas of some kidney glomeruli and nephrotic range proteinuria [1,2]. While corticosteroids or calcineurin inhibitors can ameliorate the disease, FSGS is frequently resistant to treatment and may progress to end stage renal disease (ESRD) [1,3]. The cell type mainly affected and causing FSGS is the podocyte (visceral glomerular epithelial cell). In healthy individuals, interdigitating podocyte foot processes together with the basal lamina and underlying, fenestrated endothelial cells form a sieve-like structure thereby enabling the filtration of blood. A high number of specific genes is necessary to maintain the complex three-dimensional structure of podocytes. The impacts initiating FSGS are heterogeneous and include gene mutations, circulating factors, drugs, viruses and hypertension [1,2]. These very different etiologies lead to a common final path characterised by foot process effacement and loss of podocytes concomitant with nephrotic syndrome, fibrosis and scarring.

Many FSGS-causing mutations have been identified so far [4], but for the majority of patients other causes seem to be responsible and the exact molecular changes are still widely elusive. In order to develop novel therapeutic approaches suitable for a high amount of patients, a better characterisation of the common molecular events in the final path seems to be of high relevance. Several studies addressed gene expression changes in FSGS patients giving insight on the glomerular level, which unfortunately does not allow for a detailed understanding of podocyte-specific events [5–7].

To overcome this limitation, Tobias Huber and coworkers established a protocol to isolate GFP-marked mouse podocytes by FACS sorting and determined the set of expressed genes in healthy podocytes [8]. A similar system was also used by the Potter group to determine specific changes in podocytes, mesangial cells and endothelial cells in *Cd2ap*^-/—^and *Cd2ap*^+/-^;*Fyn*^-/—^driven FSGS [9,10].

In another approach, by translating ribosome affinity purification (TRAP) the group of Benjamin Humphreys has managed to characterize gene expression changes in podocytes upon *Actn4* KO [11]. Unexpectedly, in none of these FSGS models, classical podocyte marker genes (e.g. *Wt1*, *Nphs1*, *Nphs2*, *Podxl*) significantly changed. Therefore, any comparison of gene expression changes during FSGS on the glomerular level runs at high risk of only assessing the changes based on podocyte loss but not the changes *within* podocytes. These data furthermore raised the question if dysregulation of classical podocyte marker genes in FSGS was generally not very pronounced or just in the models analysed.

Thus, we addressed these issues in the *miR-193a* FSGS model [12] and tried to identify novel biomarkers and therapeutic targets for FSGS.

## Materials and methods

### Animal experiments

*miR-193a* and Gt(ROSA)26^Sortm4(ACTB-tdTomato,-EGFP)Luo/J^ (miR-193a) x *hNPHS2*Cre (podGFP) mice and their handling were described before [8,12]. Genotyping primers were CGATCAGGATGATCTGGACG and CAAGCTCTTCAGCAATATCAC (for *miR-193a*) and CTCTGCTGCCTCCTGGCTTCT and TCAATGGGCGGGGGTCGT (for Tomato). The two lineages were crossed to obtain *miR-193a*-overexpressing *GFP*-positive podocytes (*miR-193a* x *GFP*), while their podGFP littermates without transgenic *miR-193a* construct served as controls (wt x podGFP). *miR-193a* was induced by 1 mg/ml doxycycline in 5% sucrose, podGFP mice fed with doxycycline solution served as a control. 15 weeks old females were used for this study. Anaesthesia was performed with 100mg/kg Ketamine and 5mg/kg Xylazine i.p. Mice were sacrificed by cervical dislocation. All animal experiments and handling were in accordance with the Austrian law for protection of animals and approved by the animal ethics committee of the Austrian ministry for science and research (66.009/0053-II/3b/2014). AFOG analysis was performed according to a standard protocol. Urinary albumin levels were assessed by ELISA (E90-134, Bethyl Laboratories, Montgomery, TX, USA). Creatinine levels were measured with the Creatinine Assay Kit (STA-378, Cell Biolabs, San Diego, CA, USA).

### Human samples

Human data were obtained from the fusion of dysregulated genes found in two independent published studies [5,6]. In short, in the first study [5], all 4 patients suffered from idiopathic FSGS and were female. Urinary protein levels were 4.0, 5.4, 14.7, and 17.0 (g/day) and serum creatinine levels were 0.8, 1.1, 0.9, and 5.3 (mg/dl), respectively. Controls were obtained from ‘normal’ regions of kidneys removed from Wilms’ tumor patients. In the second study [6], FFPE renal biopsy material from 19 patients with idiopathic nephrotic syndrome (edema, proteinuria >3.5 g/day; serum albumin <3.0 g/dl) and 2 without full nephrotic syndrome was used. Controls were renal biopsies that appeared normal by histological and electron microscopic examination obtained from renal biopsies performed for minimal isolated proteinuria or hematuria (seven patients) or tissue from uninvolved portions of a kidney at the time of tumor nephrectomies.

### Cell isolation, RNA isolation and qPCR

Podocytes were isolated as described before from 4 animals/group [8]. RNA was isolated with the ReliaPrep RNA kit from Promega according to manufacturer’s instructions. qPCRs for *Arntl*, *Bhlhe41*, *Cdh11*, *Dbp*, *EphA6*, *Ggt*, *Mt2*, *Per2*, *Per3*, *Prss23*, *S100a6*, *Spns2* and Cyclophilin B (for normalisation) were performed on a CFX96 Real Time System with a C1000 Thermal Cycler (Bio-Rad) using KAPA SYBR FAST from Sigma Aldrich. Primer sequences were from PrimerBank and were TGACCCTCATGGAAGGTTAGAA and GGACATTGCATTGCATGTTGG (*Arntl*, forward and reverse), TGTGTAAACCCAAAAGGAGCT and TGTTCGGGCAGTAAATCTTTCAG (*Bhlhe41*), CTGGGTCTGGAACCAATTCTTT and GCCTGAGCCATCAGTGTGTA (*Cdh11*), GGAAACAGCAAGCCCAAAGAA and CAGCGGCGCAAAAAGACTC (*Dbp*), TGCGAAGTCCGGGAATTTCTT and GCAACACAACTTGGTTGGAGAC (*EphA6*), TTCAATGGGACAGAAACCTTGAG and TCCCTGTGTATAAGACCTCCG (*Ggt5*), GCCTGCAAATGCAAACAATGC and AGCTGCACTTGTCGGAAGC (*Mt2*), GAAAGCTGTCACCACCATAGAA and AACTCGCACTTCCTTTTCAGG (*Per2*), AACACGAAGACCGAAACAGAAT and CTCGGCTGGGAAATACTTTTTCA (*Per3*), GGTGAGTCCCTACACCGTTC and GGCGTCGAAGTCTGCCTTAG (*Prss23*), ATTGGCTCCAAGCTGCAGG and TCATTGTAGATCAAAGCCAAGG (*S100a6*), CCATCCTGAGTTTAGGCAACG and GATCACCTTTCTATTGAAGCGGT (*Spns2*).

### RNAseq

RNAseq was performed by the NGS unit of the Vienna Biocenter Core Facilities GmbH (VBCF) (www.viennabiocenter.org/facilities). For RNA preparation we used NEBNext Ultra 2 kit (New England Biolabs; https://international.neb.com/products/e7645-nebnext-ultra-ii-dna-library-prep-kit-for-illumina). The samples were paired end sequenced with a read length of 50 base pairs on an Illumina HiSeq2500 (V4 chemistry) according to the manufacturer’s protocol (RTA 1.18.66.3). RNA-Seq reads were mapped to the reference genome using TopHat [13] and the differential gene expression analysis was calculated by CuffDiff v2.2.1 using blind dispersion method [14].

### Statistics

Due to low total RNA yield in podocytes, RNA of 4 mice was pooled for RNAseq making it impossible to calculate p-values of the results for independent mice. For qPCR, results of one of two independent experiments with three wild-type and three miR-193a FSGS mice are shown. Statistical significance was calculated by two-tailed Student’s t-test.

## Results

**Fig 1:**
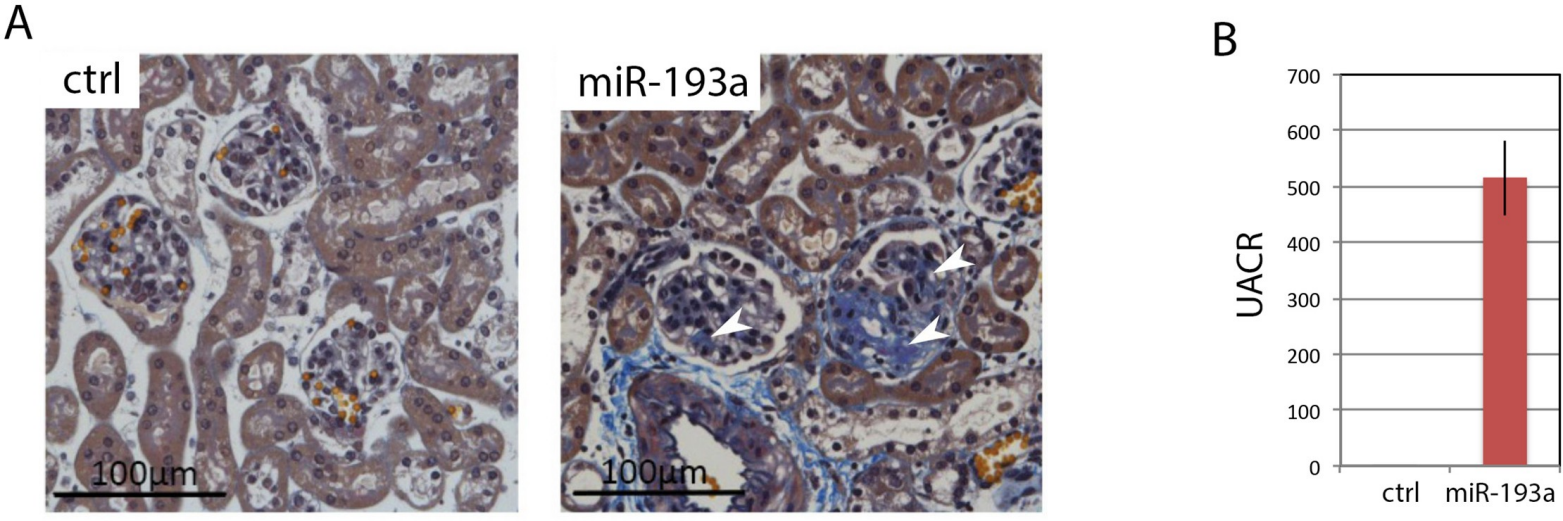
*miR-193a*-driven FSGS. A) Histology of *miR-193a* and control mice 8.5 weeks post FSGS induction. Acid Fuchsin Orange G staining, 400x magnification, scale bars represent 100μm. B) Corresponding UACR of miR-193a and control mice. UACR, urinary albumin:creatinin ratio.

*miR-193a* suppresses *Wt1*, a master regulator of podocyte function, thereby causing FSGS [12]. We crossed *miR-193a* mice with Gt(ROSA)26^Sortm4(ACTB-tdTomato,-EGFP)Luo/J^ x *hNPHS2*Cre mice to obtain GFP-tagged podocytes with inducible *miR-193a*, while GFP-tagged podocytes from littermates without *miR-193a* construct served as control [8,12]. Irreversible podocyte loss and FSGS were initiated by doxycycline-driven overexpression of *miR-193a* for 7 weeks, followed by 10 days without doxycycline (Fig 1). This allowed us to focus on transcript changes directly related to FSGS and neglect changes only related to increased *miR-193a*. Podocytes from FSGS and control mice were isolated by FACS sorting according to an established protocol [8] and submitted to RNAseq analysis.

S1 Table depicts the expression changes upon *miR-193a*-induced FSGS. The strongest upregulation for a protein-coding gene was found for the trans-membrane receptor tyrosine kinase Ephrin receptor A6 (*EphA6*), which integrates extra-cellular signals and has never been linked to FSGS before. Ephrin receptors can bind to *Nck* and *Src*, which both have been implicated in FSGS [15]. Among the strongly upregulated genes were also the metallothioneins *Mt1* and *Mt2*, which can interfere with FSGS and diabetic nephropathy [16,17]. Their upregulation might represent a cell-autonomous mechanism to attenuate FSGS progression. Obscurin (*Obscn*; *Arhgef30*; Cytoskeletal Calmodulin And Titin-Interacting RhoGEF), also upregulated and never associated with FSGS, is a modulator of the cytoskeleton and activator of *RhoA* [18,19] and might therefore be able to induce FSGS [20]. We also found strongly increased levels of Serine Protease 23 (*Prss23*) and Sphingolipid Transporter 2 (*Spns2*), which has been associated with kidney fibrosis before [21]. Strikingly, the transcripts of several circadian genes, including Basic Helix-Loop-Helix Family Member E41 (*Bhlhe41*, *Dec2*), the Period Circadian Regulators 2 and 3 (*Per2*, *Per3*) and D Site Of Albumin Promoter Binding Protein (*Dbp*) were massively enhanced. In line with this, the protein-coding gene with the strongest downregulation was Aryl Hydrocarbon Receptor Nuclear Translocator Like (*Arntl*, *Bmal*, *Bhlhe5*), which is a circadian master regulator repressed by *Per2*. *Arntl* is associated with susceptibility to hypertension and diabetes and can activate the TNF receptor Osteoprotegerin [22–24]. Another strongly downregulated gene was Cadherin 11 (*Cdh11*), a calcium-dependent mediator of cell adhesion and cytoskeleton and inhibitor of Wnt and Rho activation [25].

**Fig 2:**
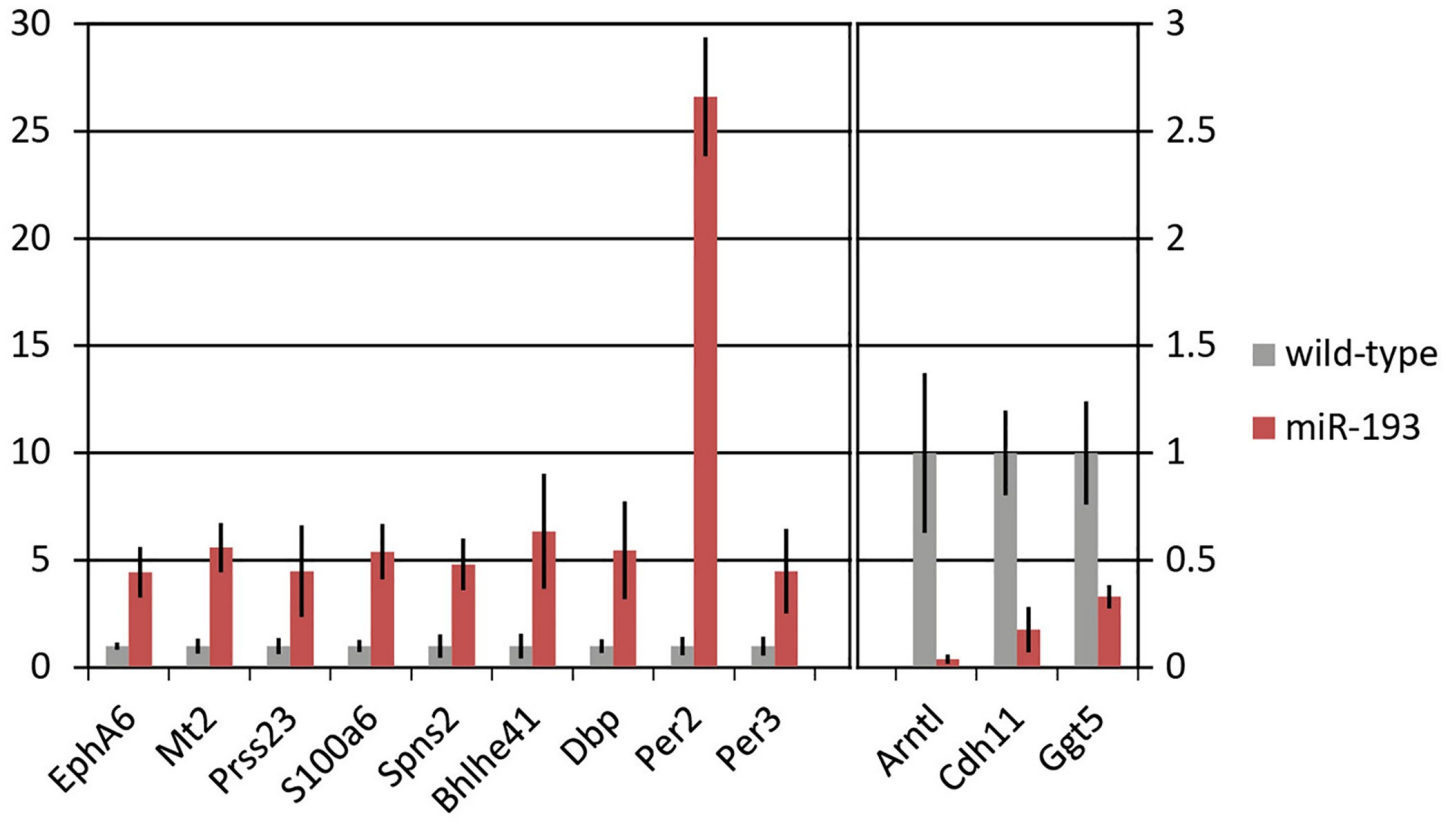
Dysregulated genes in podocytes of *miR-193a* FSGS mice. Expression changes of selected genes upon *miR-193a*-driven FSGS in isolated podocytes were assessed by qPCR and normalised to *CycB*. Results are representative medium values (mean±SEM) of three independent mice of one of two independent experiments. Control was set to 1. *Arntl*, Aryl Hydrocarbon Receptor Nuclear Translocator Like; *Bhlhe41*, Basic Helix-Loop-Helix Family Member E41; *Cdh11*, Cadherin 11; *CycB*, cyclophilin B; *Dbp*, D Site Of Albumin Promoter Binding Protein; *EphA6*, EPH Receptor A6; *Ggt5*, Gamma-Glutamyltransferase 5; *Mt2*, Metallothionein 2; *Per2*, Period Circadian Regulator 2; *Per3*, Period Circadian Regulator 3; *Prss23*, Serine Protease 23; *S100a6*, S100 Calcium Binding Protein A6; *Spns2*, Sphingolipid Transporter 2; *p < 0.025; **p < 0.005.

Table 1 depicts the 20 strongest up- and downregulated genes identified in the *miR-193a* model according to RNAseq. We confirmed several of the dysregulated genes by qPCR (Fig 2).

**Table 1:** The 20 strongest up- and downregulated genes in podocytes during *miR193a*-driven FSGS compared to wild-type as assessed by RNAseq. FC, fold change.

Symbol	FC (log2)	Name
Epha6	8.4	EPH Receptor A6
Bhlhe41	5.8	Basic Helix-Loop-Helix Family Member E41
Adrb2	5.4	Adrenoceptor Beta 2
Mt2	4.7	Metallothionein 2
Dbp	4.7	D-Box Binding PAR BZIP Transcription Factor
Ttll7	4.5	Tubulin Tyrosine Ligase Like 7
Abcc3	4.5	ATP Binding Cassette Subfamily C Member 3
Osbpl6	4.4	Oxysterol Binding Protein Like 6
Pnpla7	4.1	Patatin Like Phospholipase Domain Containing 7
A2bp1	4.0	RNA Binding Fox-1 Homolog 1
Pcdh1	4.0	Protocadherin 1
Exdl1	3.9	Exonuclease 3'-5' Domain Containing 1
Obscn	3.9	Obscurin, Cytoskeletal Calmodulin And Titin-Interacting RhoGEF
Gdnf	3.8	Glial Cell Derived Neurotrophic Facto
Gng10	3.8	G Protein Subunit Gamma 10
Zbtb16	3.8	Zinc Finger And BTB Domain Containing 16
Pik3ip1	3.8	Phosphoinositide-3-Kinase Interacting Protein 1
Rcan2	3.7	Regulator Of Calcineurin 2
Garnl3	3.7	GTPase Activating Rap/RanGAP Domain Like 3
Per2	3.7	Period Circadian Regulator 2
Arntl	-5.6	Aryl Hydrocarbon Receptor Nuclear Translocator Like
Rtf1	-5.2	RTF1 Homolog, Paf1/RNA Polymerase II Complex Component
Adra2c	-4.9	Adrenoceptor Alpha 2C
Lmbr1	-4.0	Limb Development Membrane Protein 1
Nlrc5	-4.0	NLR Family CARD Domain Containing 5
Kcnh3	-3.9	Potassium Voltage-Gated Channel Subfamily H Member 3
Ccdc88c	-3.8	Coiled-Coil Domain Containing 88C
Rai2	-3.7	Retinoic Acid Induced 2
Amy1	-3.5	Amylase Alpha 1A
Sp6	-3.4	Sp6 Transcription Factor
Pim1	-3.4	Pim-1 Proto-Oncogene, Serine/Threonine Kinase
Ccnjl	-3.4	Cyclin J Like
Spon1	-3.3	Spondin 1
Dll1	-3.2	Delta Like Canonical Notch Ligand 1
Hoxd9	-3.2	Homeobox D9
Cdc7	-3.1	Cell Division Cycle 7
Bcl2l11	-3.0	Bcl-2-Like Protein 11
Rims1	-3.0	Regulating Synaptic Membrane Exocytosis 1
Obsl1	-2.9	Obscurin Like Cytoskeletal Adaptor 1
Unc5b	-2.9	Unc-5 Netrin Receptor B

**Fig 3:**
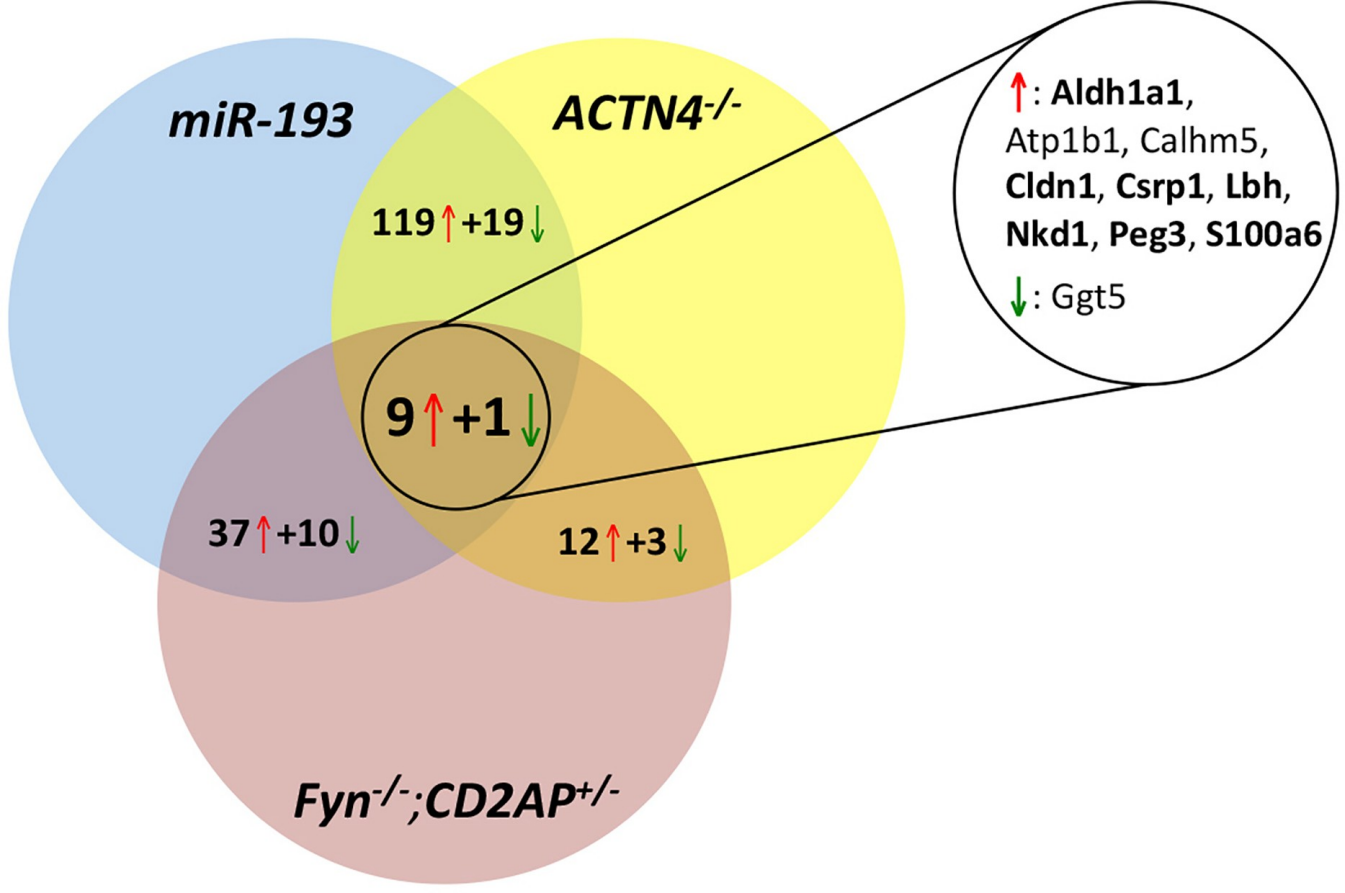
Dysregulated genes in podocytes in different FSGS models. An overlap of three independent mRNA expression profiles (*miR-193*, *Actn4*^-/-^, Fyn^-/-^;*Cd2ap*^+/-^,) during FSGS reveals nine commonly upregulated genes (red arrow) and one downregulated gene (green arrow). Seven genes (bold font) are associated with the Wnt signaling pathway. *Aldh1a1*, Aldehyde Dehydrogenase 1 Family Member A1; *Atp1b1*, ATPase Na+/K+ Transporting Subunit Beta 1; *Calhm5*, Calcium Homeostasis Modulator Family Member 5; *Cldn1*, Claudin 1; *Csrp1*, Cysteine And Glycine Rich Protein 1; *Lbh*, LBH Regulator Of Wnt Signaling Pathway; *Nkd1*, Nkd Inhibitor Of Wnt Signaling Pathway 1; *Peg3*, Paternally Expressed 3; *S100a6*, S100 Calcium Binding Protein A6; *Ggt5*, Gamma-Glutamyltransferase 5.

In order to determine which of the detected changes were not only specific for our system, but represented more general changes during FSGS we compared them with data from *Actn4*^-/—^and *Cd2ap*^+/-^;*Fyn*^-/—^driven FSGS [10, 11]. Nine genes were up- and one gene was downregulated in all 3 models (Fig 3). Strikingly, seven (*Aldh1a1*, *Cldn1*, *Csrp1*, *Lbh*, *Nkd1*, *Peg3*, *S100a6*) of the nine genes upregulated in all three models were associated with the Wnt signaling cascade [26–34] (Fig 3). We found an especially strong overlap between the *miR-193a* and the *Actn4*^-/-^ model, suggesting that several of the genes identified by us and Grgic *et al*. [11] might be of general relevance for FSGS. In line with this, 16 of the top 20 upregulated genes in the *Actn4* KO were also strongly increased in our screen (S2 Table) [11].

**Fig 4:**
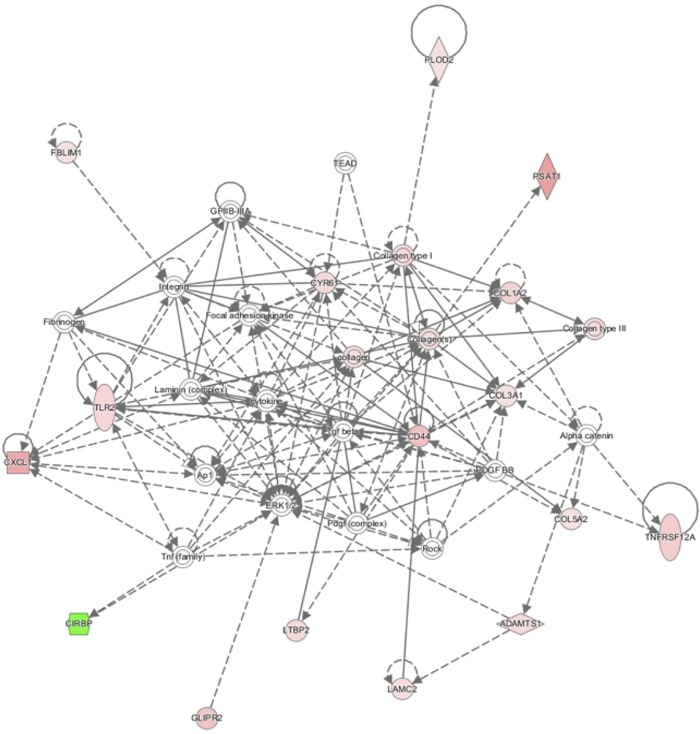
A core set of FSGS genes. The network of 35 genes as core set of FSGS identified by Ingenuity Pathway Analysis (Qiagen).

To predict which podocyte-specific changes might occur in human FSGS, we overlapped the at least two-fold dysregulated genes of our screen with the set of at least two-fold dysregulated genes in glomeruli of patients suffering from idiopathic FSGS, as podocytes cannot be isolated from human in bigger quantities [5,6]. While the comparison of genes dysregulated in isolated mouse FSGS podocytes and human FSGS glomeruli bears the risk of missing some changes due to loss of podocytes from the glomeruli during FSGS, we believe that we are still able to define several *bona fide* podocytic human FSGS genes, especially amongst the upregulated genes. The identified genes included several already associated with FSGS (e.g. *Col1a1*, *Col1a2*, *Cd24*, *Cd44*, *Plau*, *Umod*) and many novel genes not associated with FSGS so far (S3 Table). To narrow down this list even further we overlapped the genes dysregulated in human disease with both the at least two-fold dysregulated genes in the *miR-193a* model and in the *Actn4* KO. This led to the definition of a 35-gene-set which might constitute a core set of podocyte-specific FSGS genes (Table 2). Strikingly, this set contains 17 genes related to ECM modifications, 8 genes closely related to metabolism, and 4 genes related to transmembrane transport. This enrichment in ECM-related genes differs strongly from the most abundant processes in healthy podocytes, namely cytoskeleton regulation and protein transport [8]. Analysis of GO term enrichment also showed collagen fibril organisation/ECM organisation as the top enriched process (Fig 4).

**Table 2:** An overlap of glomerular changes in human FSGS, and podocytic changes in *Actn4*-KO- and *miR-193a*-induced FSGS identifies a core set of 35 commonly dysregulated genes.

Symbol	Name	context	change
ADAMTS1	ADAM Metallopeptidase With Thrombospondin Type 1 Motif 1	ECM	up
BACE2	Beta-Secretase 2	ECM	up
CD44	CD44 Molecule	ECM, cell-cell interactions	up
COL1A2	Collagen Type I Alpha 2 Chain	ECM	up
COL3A1	Collagen Type III Alpha 1 Chain	ECM	up
COL5A2	Collagen Type V Alpha 2 Chain	ECM	up
CXCL1	C-X-C Motif Chemokine Ligand 1	ECM	up
CYR61	Cellular Communication Network Factor 1	ECM	up
FN1	Fibronectin 1	ECM	up
GLIPR2	GLI Pathogenesis Related 2	ECM	up
LAMC2	Laminin Subunit Gamma 2	ECM	up
LTBP2	Latent TGF Beta Binding Protein 2	ECM	up
PLOD2	Procollagen-Lysine,2-Oxoglutarate 5-Dioxygenase 2	ECM	up
PRSS23	Serine Protease 23	ECM	up
SLIT3	Slit Guidance Ligand 3	ECM	up
THBD	Thrombomodulin	ECM	up
TNFRSF12A	TNF Receptor Superfamily Member 12A	ECM, inflammation	up
ALDH18A1	Aldehyde Dehydrogenase 18 Family Member A1	metabolism	up
ALDH1A1	Aldehyde Dehydrogenase 1 Family Member A1	metabolism	up
ASRGL1	Asparaginase And Isoaspartyl Peptidase 1	metabolism	up
ELOVL7	ELOVL Fatty Acid Elongase 7	metabolism	up
GUCY1A3	Guanylate Cyclase 1 Soluble Subunit Alpha 1	metabolism	up
PPP1R3C	Protein Phosphatase 1 Regulatory Subunit 3C	metabolism	up
PSAT1	Phosphoserine Aminotransferase 1	metabolism	up
PTPLAD2	3-Hydroxyacyl-CoA Dehydratase 4	metabolism	up
ABCC4	ATP Binding Cassette Subfamily C Member 4	transport	up
ATP1B1	ATPase Na+/K+ Transporting Subunit Beta 1	transport	up
SLC1A4	Solute Carrier Family 1 Member 4	transport	up
SLCO2B1	Solute Carrier Organic Anion Transporter Family Member 2B1	transport	up
CLDN1	Claudin 1	cell contact, slit diaphragm	up
FBLIM1	Filamin Binding LIM Protein 1	cytoskeleton	up
TLR2	Toll Like Receptor 2	inflammation	up
MAFF	MAF BZIP Transcription Factor F	transcription	up
CIRBP	Cold Inducible RNA Binding Protein	survival	down
NRIP2	Nuclear Receptor Interacting Protein 2	signalling	down

## Discussion

In this study we tried to analyse transcriptional changes in podocytes during FSGS in the *miR-193a* mouse model and identified many genes not associated with FSGS so far. While the RNAseq analysis was performed in pooled samples from four mice per group due to the low yield of podocyte RNA per mouse, we were able to confirm the observed dysregulation for a considerable number of selected genes by qPCR in individual mouse samples (Fig 2) and our data have a good overlap with the data by Grgic et al. [11; S2 Table], which makes us confident that our findings are of relevance.

We observed a massive dysregulation of circadian genes in the *miR-193a* FSGS model. It is known that circadian genes are crucially involved in the regulation of inflammation, immune response and kidney physiology and disease including glomerular diseases [35,36]. While melatonin has been shown to ameliorate chronic kidney disease via antioxidant effects and modulation of the renin-angiotensin system [37], the detailed interactions between the circadian clock and glomerular diseases are not elucidated and will be a highly interesting field for follow-up studies.

A comparison of our results with published data in other model systems revealed a dysregulation of genes associated with the Wnt cascade as common feature in FSGS. Claudin 1 (*Cldn1*) and S100 Calcium Binding Protein A6 (*S100a6*) are β-Catenin targets that can reciprocally activate β-Catenin [26–29]. Enhanced expression of Claudin 1 can induce proteinuria through slit diaphragm destabilisation [38]. Aldehyde Dehydrogenase 1 Family Member A1 (*Aldh1a1*) and Limb Bud And Heart Development (*Lbh*) have been shown to be upregulated by β -Catenin and control cell differentiation [30,31]. *Aldh1a1* is able to regulate the differentiation and repair process of podocytes during injury via retinoic acid production [39]. Cysteine and glycine-rich protein 1 (*Csrp1*) and Naked Cuticle Homolog 1 (*Nkd1*) modulate Wnt signaling and cytoskeletal rearrangements by interaction with Dishevelled [32,33]. The imprinted gene Paternally Expressed 3 (*Peg3*) is an inhibitor of Wnt signaling [34]. Activation of the Wnt/sβ -Catenin cascade in FSGS patients has also been observed by others [40,41] suggesting that targeting Wnt signalling might be a therapeutic option in FSGS [42].

By overlapping podocyte-specific changes in FSGS models with glomerular data from human patients we found a set of 35 commonly dysregulated genes. Strikingly, this set exhibited a strong enrichment in ECM-associated genes. Of note, it is not the mere loss of functional, differentiated podocytes, but progressive glomerular sclerosis that eventually leads to ESRD. In line with this, several mutations in ECM-associated, FSGS-causing genes have been described (Collagen 4, Laminin B2, the integrins α-4 and β-3, *ApoL1*, and *Cd151*) and the circulating factor *suPAR* is suggested to promote FSGS by modulation of β-3 integrin signaling [4,43]. Therefore, this shared gene set with almost 50% of ECM-associated genes might be of interest for the development of novel therapeutic concepts.

The second big group consisted of 8 genes related to an altered metabolism and might be a consequence of the podocytic attempts to morphologically adjust to loss of podocyte differentiation and number. Stress-related podocyte growth and hypertrophy is a well-known observation during FSGS also receiving a lot of attention recently [44,45].

Of note, only two of the 35 genes were downregulated, while 33 were upregulated. It is a general observation in FSGS profilings, that the majority of genes is upregulated [5,6,10,11], possibly due to gene reactivation during dedifferentiation.

Furthermore, in all three different FSGS models (*miR-193a*, *Actn4* KO, *Cd2ap*^+/-^;*Fyn*^-/-^) classical podocyte marker genes did not appear strongly changed, with the exception of respective model-specific, disease-causing alterations. This suggests that our current understanding of podocyte biology during FSGS might deserve reconsideration. While these findings might describe the general changes during FSGS relevant for disease progression, we cannot exclude that additional podocyte populations with distinct gene expression signatures exist as FSGS is focal and segmental and injured podocytes are frequently lost before they can be isolated [46]. A thorough analysis of the identified genes and their potential as therapeutic targets should be the focus of follow-up studies.

## Supporting information

S1 Data (XLSX)

S1 Table Gene expression changes in isolated podocytes upon miR193a-induced FSGS as compared to wild-type. (XLSX)

S2 Table Comparison of the top 20 upregulated genes in Actn4-KO- with miR-193a-induced FSGS reveals a strong overlap. (XLSX)

S3 Table Shared sets of dysregulated genes identified by comparision of mouse podocytes of miR193a-induced FSGS and glomeruli of human FSGS biopsies. (XLSX)
